# IgG and IgM responses to PfEMP1 domains associated with semi-immunity to clinical malaria in Burkinabe children under five

**DOI:** 10.3389/fimmu.2026.1781670

**Published:** 2026-04-02

**Authors:** Benedicta O. Dankyi, Takaaki Yuguchi, Rattanaporn Rojrung, Hikaru Nagaoka, Bernard N. Kanoi, Alfred B. Tiono, Issa Nebie, Alphonse Ouedraogo, Keiko Tanaka, Yoshihiro Miyake, Kazutoyo Miura, Jetsumon Sattabongkot, Sodiomon B. Sirima, Takafumi Tsuboi, Eizo Takashima

**Affiliations:** 1 Division of Malaria Research, Proteo-Science Center, Ehime University, Matsuyama, Japan; 2 Centre for Malaria Elimination, Institute of Tropical Medicine, Mount Kenya University, Thika, Kenya; 3 Groupe de Recherche Action en Santé (GRAS), Ouagadougou, Burkina Faso; 4 Department of Epidemiology and Public Health, Ehime University Graduate School of Medicine, Toon, Japan; 5 Vaccine Research Center, National Institute of Allergy and Infectious Diseases, National Institutes of Health, Bethesda, MD, United States; 6 Mahidol Vivax Research Unit, Faculty of Tropical Medicine, Mahidol University, Bangkok, Thailand; 7 Division of Cell-Free Sciences, Proteo-Science Center, Ehime University, Matsuyama, Japan

**Keywords:** AlphaScreen, antibody-mediated protection, malaria immunity, PfEMP1, *Plasmodium falciparum*, wheat germ cell-free system

## Abstract

**Background:**

Malaria remains a major global health burden, with children under five years in sub-Saharan Africa disproportionately affected. While immunity develops with repeated exposure, the specific correlates of protection in early childhood are not well defined. To address this, we assessed IgG and IgM antibody responses to *Plasmodium falciparum* erythrocyte membrane protein 1 (PfEMP1) domains in plasma samples from 80 Burkinabe children (aged 0–60 months) collected before (pre-malaria season) and during peak malaria transmission.

**Methods:**

Using AlphaScreen, we measured antibody responses to 271 PfEMP1 (3D7) domains expressed via wheat germ cell-free synthesis.

**Results:**

Pre-season analysis showed that 96% of domains were IgG-reactive and IgG breadth increased with age, whereas 71% were IgM-reactive, but IgM breadth showed no age trend. IgG responses to 132 domains (49%) remained significantly associated with reduced risk of clinical malaria after false discovery rate (FDR) correction, including many non-adhesion domains. Five (5) IgM responses were nominally protective, but none remained significant after FDR correction. Fc-dependent opsonic phagocytosis assay using a subset of the top-ranked domains revealed that opsonic phagocytosis activity correlated with protection for only one antigen, suggesting that PfEMP1-specific antibodies may mediate protection through mechanisms beyond phagocytosis in children under 5 years old.

**Conclusions:**

This study provides the first comprehensive characterization of IgG and IgM responses to a large panel of PfEMP1 domains in young children, revealing broad antibody recognition and selective opsonic phagocytosis activity. Findings from the study deepen our understanding of early-life immunity to malaria and help identify PfEMP1 regions of interest for future vaccine development.

## 1 Introduction

Malaria continues to be a significant global health challenge, claiming more than half a million lives in 2024, with over 90% of these deaths occurring in the WHO African region. Tragically, children under five account for the largest share of malaria-related mortality (1). As maternally derived antibodies wane around 4–6 months of age, infants become highly susceptible to *Plasmodium falciparum* infection (2) and gradually acquire immunity through repeated exposure (3). Understanding the immune mechanisms that mediate early protection is essential for guiding vaccine development (4).

Antibodies targeting variant surface antigens (VSAs) on infected erythrocytes are known contributors to naturally acquired immunity (5). Among these, *P. falciparum* erythrocyte membrane protein 1 (PfEMP1) is a key virulence factor that enables cytoadhesion and immune evasion (6). Antibodies to PfEMP1, especially those directed at its Duffy-binding-like (DBL) and Cysteine-rich Interdomain Region (CIDR) domains, have been associated with protection against clinical malaria (7–9). Consistent with this, recent work in Malian children shows that high antibody levels to selected PfEMP1 domains are associated with reduced parasite burden and enhanced opsonic phagocytic activity, supporting a role for domain-specific antibody responses in early-life immunity (10). However, little is known about how domain-specific antibody responses develop in children under five, who represent the highest-risk group for severe disease.

Although IgG has been the central focus of malaria immunology, emerging evidence highlights an important but understudied role for IgM (11–14). Boyle et al. (12) found that higher merozoite-specific IgM levels correlate with reduced malaria risk in children, while Chan et al. (15) showed that IgM contributes to transmission-blocking immunity. Arama et al. (11) further demonstrated that the malaria-resistant Fulani population mounts strong IgM responses to diverse *P. falciparum* antigens, including those linked to protection. These results suggest that IgM may play a more substantial role in protective immunity than previously appreciated (16).

Recent advances in antigen discovery have benefited from the use of high-throughput protein arrays. The wheat germ cell-free system (WGCFS) offers major advantages for expressing large, complex, non-glycosylated malaria proteins in near-native conformations without codon optimization (17, 18). Proteins expressed using WGCFS show enhanced immunoreactivity to human sera compared to those produced via *E. coli* expression systems (19–22). In parallel, the AlphaScreen platform enables sensitive detection of antibody-antigen interactions without protein purification or immobilization (8), making it a valuable tool for high-throughput immunoprofiling and antigen discovery (23).

Antibody-mediated effector functions play a central role in naturally acquired immunity to *P. falciparum.* These functions are mediated through multiple complementary pathways, including neutralization, opsonization for phagocytosis, and recruitment of cellular effectors through Fc receptors (24, 25). Among these, opsonic phagocytosis via Fcγ receptor engagement on monocytes and macrophages has been associated with clinical protection in malaria-endemic populations (26, 27). Antibody-dependent cellular cytotoxicity (ADCC) mediated by natural killer (NK) cells represents another Fc-dependent mechanism of malaria immunity (28). In addition to Fc-dependent ADCC mediated by PfEMP1-specific IgG, certain PfEMP1 domains can also directly activate NK cells through Fc-independent interactions with natural cytotoxicity receptors, including NCR3 (NKp30) (29, 30).

Fcγ receptor-mediated opsonic phagocytosis is a well-established functional readout of antibody activity, particularly in studies of early-life immunity (27, 31). However, the functional activity of antibodies targeting individual PfEMP1 domains remains poorly understood, especially in children under five years of age. To address these knowledge gaps, we investigated IgG and IgM responses to 271 PfEMP1 domains in Burkinabe children under five years of age. We hypothesized that young children generate early PfEMP1-specific antibody responses that correlate with protection from clinical malaria. PfEMP1 domains from the 3D7 reference strain were expressed using WGCFS, and antibody responses were quantified using AlphaScreen assay in plasma collected during the pre-malaria transmission season. To validate serological associations, we performed opsonic phagocytosis assay using THP-1 monocytes as one of the functional markers of protection and selected PfEMP1 domains associated with protection. By integrating sero-epidemiological profiling with opsonic phagocytosis assay, this study provides mechanistic insights into early-life PfEMP1 immunity and identifies domain-specific antibody responses associated with protection.

## 2 Materials and methods

### 2.1 Ethics statement

The study protocol and informed consent procedures were approved by the Burkina Faso Ministry of Health Ethics Committee and conducted in accordance with the International Conference on Harmonization Good Clinical Practices, the Declaration of Helsinki, and local regulatory requirements. Written informed consent was obtained from parents or legal guardian, with an independent witness present for illiterate individuals. Ethical clearances were granted by the Burkina Faso Ethics Committee for Health Research (ID: DMID No. 06-0020). Anonymized plasma samples from healthy donors in Bangkok, a malaria non-endemic region, were provided by the Thai Red Cross under broad consent and used as negative controls. A pooled plasma sample obtained from Thai adults with prior *P. falciparum* exposure served as a positive control for the phagocytosis assay, with approvals from the Thai Ministry of Public Health and the Walter Reed Army Institute of Research (WRAIR 778; April 14, 2000) (32). Plasma samples from Japanese participants in the Aidai Cohort Study (AICOS) were used as negative controls in the phagocytosis assay, with ethics approval from the Ehime University Graduate School of Medicine (ID: Aidaiibyourin1504012) (33). The protocol for all the serum/plasma samples used was approved by the Institutional Review Board of Ehime University Hospital, Japan (ID: Aidaiibyourin 1507005).

### 2.2 Study site and sample collection

Plasma samples were obtained from Burkinabe children aged 0–5 years, as previously described (34). The study was conducted from January 2007 to early 2008 in the Saponé Health District, Bazèga Province, central Burkina Faso, an area with a marked rainy season (June-September) and dry season (October-May). Malaria transmission peaks during the rainy season, with an estimated entomological inoculation rate (EIR) ranging from 0.3 infective bites per person per month in the dry season to 44.4 during the rainy season (35). At the time of the study, seasonal malaria chemoprevention (SMC) had not yet been implemented, insecticide-treated bed net (ITN) coverage was very low (<5%), and no mass drug administration or treatment of asymptomatic infections was conducted in the cohort (34). Children were monitored both actively and passively for malaria, defined as fever (>37.5°C) or symptoms with a positive smear. Uncomplicated cases were treated with Coartem^®^ or Artesunate-Amodiaquine, applying a 28-day censoring period after each episode to avoid double-counting. At the time of sample collection and follow-up, none of the children had received a malaria vaccine, as the study (34) preceded the availability and recommendation of malaria vaccines, RTS,S/AS01 and R21/Matrix-M. Plasma samples were collected longitudinally from each participant at two time points: pre-transmission (March-May 2007), and during peak-transmission (June-October 2007), as detailed in Ouédraogo et al. (34). However, the current manuscript focuses primarily on the pre-malaria season samples.

### 2.3 Production of *P. falciparum* proteins and measurement of antibody (IgG and IgM) levels with AlphaScreen assay

A library of 271 PfEMP1 domains (163 DBL and 108 CIDR) derived from 62 var genes of the 3D7 strain was produced using the wheat germ cell-free system (WGCFS) as previously described (7, 36) (**Supplementary Table 1**). Each recombinant domain was mono-biotinylated via BirA. Antigen-specific IgG and IgM responses to these proteins were quantified by AlphaScreen following the method of Yuguchi et al. (37) with minor modifications. All liquid handling steps were semi-automated using VIAFLO 384 and VIAFLO ASSIST (Integra Biosciences, Zizers, Switzerland). For each reaction, 5 µL of 50-fold-diluted, non-purified biotinylated PfEMP1 protein was mixed with 10 µL of 4000-fold-diluted plasma in reaction buffer (100 mM Tris-HCl pH 8.0, 0.01% Tween-20, and 0.1% bovine serum albumin). The mixture was incubated in 384-well OptiPlates (PerkinElmer, Waltham, MA, USA) at 26°C for 30 minutes. Subsequently, 10 μL of a bead mixture containing streptavidin-conjugated donor beads and either protein G-conjugated acceptor beads (for IgG detection; Thermo Scientific, Waltham, MA, USA) or anti-human IgM-conjugated acceptor beads (for IgM detection; Jackson ImmunoResearch Laboratories, West Grove, PA, USA) was added. Plates were incubated for 60 minutes in the dark to facilitate donor bead binding to biotinylated proteins and acceptor bead binding to plasma IgG or IgM. Luminescence at 620 nm was measured on an EnVision plate reader (PerkinElmer). Serial dilutions of biotin-SP-ChomPure human IgG and IgM (Jackson ImmunoResearch Laboratories, West Grove, PA, USA) were included on each plate to generate curves and enable normalization using a 5-parameter logistic model. Plasma samples were randomized to minimize experimental bias.

Seropositivity thresholds were defined as the mean plus two standard deviations (Mean + 2SD) of log-transformed values from negative-control plasma. PfEMP1 domains were considered immunoreactive if more than 10% of participants had log-transformed, normalized AlphaScreen counts exceeding this cutoff, and only these proteins were included in subsequent analyses (37).

### 2.4 Opsonic phagocytosis assay

Antibody-mediated opsonic phagocytosis of selected recombinant PfEMP1 domains was assessed using undifferentiated THP-1 cells and flow cytometry, following Feng et al. (26) with minor modifications. Briefly, 20 µL of PfEMP1-coupled fluorescent beads (5 × 10^7^ beads/mL) (Sigma-Aldrich, Merck KGaA, Darmstadt, Germany) were incubated with 2 µL of heat-inactivated plasma (1:10 dilution, 56 °C for 30 min) for 1 h at room temperature to allow opsonization. Beads were then washed three times with RPMI-1640 and co-incubated with 100 µL of THP-1 cells (5 × 10^5^ cells/mL) (JCRB Cell Bank, Ibaraki, Japan) at 37°C, 5% CO_2_ for 45 min. Phagocytosis was terminated by centrifugation (400 × g, 3 min, 4°C), and cells were fixed in 2% paraformaldehyde before analysis by flow cytometry (CytoFLEX S, Beckman Coulter, Indianapolis, IN, USA). Data were analyzed using FlowJo® software (version 10.10.0). THP-1 cells were maintained under standard conditions in RPMI-1640 supplemented with 10% heat-inactivated fetal bovine serum. Plasma from malaria-naïve Japanese volunteers served as a negative control, while plasma from Thai adults with prior malaria exposure was included as a positive control.

### 2.5 Statistical analysis

Statistical analyses were conducted using R software (Version 4.3.2) and GraphPad Prism 8 (GraphPad Software, LCC). Normalized AlphaScreen counts were log-transformed before analysis and are referred to as ASC throughout this study.

Median seroprevalence between the CIDR and DBL domains was compared using Wilcoxon rank-sum tests, overall and within age groups. Differences in antibody breadth between the younger age group and the older age groups were also evaluated using Wilcoxon rank-sum tests. Associations between age and breadth were assessed using Spearman’s rank correlation.

Pre-season ASC associations with the number of episodes per period of malaria risk (RpT), were evaluated using Spearman’s rank correlation. RpT was calculated as the number of episodes during the follow-up period divided by the time at risk. Time at risk was defined as the total surveillance time (355 to 401 days) minus the product of episode count and the censoring duration (28 days). Thus, higher RpT values reflect an increased number of malaria episodes (37). Individuals with RpT value of 0 were excluded from RpT-based correlation analysis, as these values indicate no measurable malaria risk during follow-up. P-values were adjusted for multiple comparisons using FDR. Interdomain correlations of IgG and IgM, and correlations between IgG and IgM to the same domain, were calculated using Spearman’s correlation. Opsonic phagocytosis associations with RpT were also assessed by Spearman’s correlation test. All tests were two-sided, with p < 0.05 considered significant.

## 3 Results

### 3.1 Characteristics of the plasma samples

A total of 622 plasma samples were originally collected from 311 individuals, with each individual providing one sample during the pre-malaria season and another during the peak malaria season. From the 311 individuals, ~7 males and ~7 females (a total of 14 individuals) with at least one clinical malaria episode during the follow-up were randomly selected from each of five age groups, >0.5-1, >1-2, >2-3, >3-4, and >4–5 years. In addition, all individuals (regardless of whether they had experienced any clinical malaria episode or not) were included when they were 0-0.5 years old at the time of sample collection (n=10). As a result, 80 individuals (53.8% male, 46.2% female) were selected for the study. Although the full paired sample set is 160, the current manuscript focuses primarily on the 80 pre-malaria season samples. The cohort had a median age of 2.0 years (range: 0.2 to 4.8) with a median of 2.5 malaria episodes during follow-up (**Table 1**). The number of episodes per period of malaria risk (RpT) ranged from 0 to 0.029. Sample characteristics for the 80 individuals are summarized in **Table 1**.

**Table 1:** Characteristics of 80 individuals.

Characteristics	Number [n=80]
Age in years at pre-season sampling, median (range)	2.0 (0.2 - 4.8)
Gender (male %)	53.8
Duration of follow-up, days, median (range)	378 (355 – 401)
Days at risk, median (range)	306 (206 –337)
Number of episodes during follow up, median (range)	2.5 (0 - 6)
Participants with ≥1 malaria episode, n (%)	66 (83)
RpT, median (range)	0.008 (0 - 0.029)

### 3.2 CIDR domains are preferentially recognized by IgG but not IgM in young children

**Figure 1:**
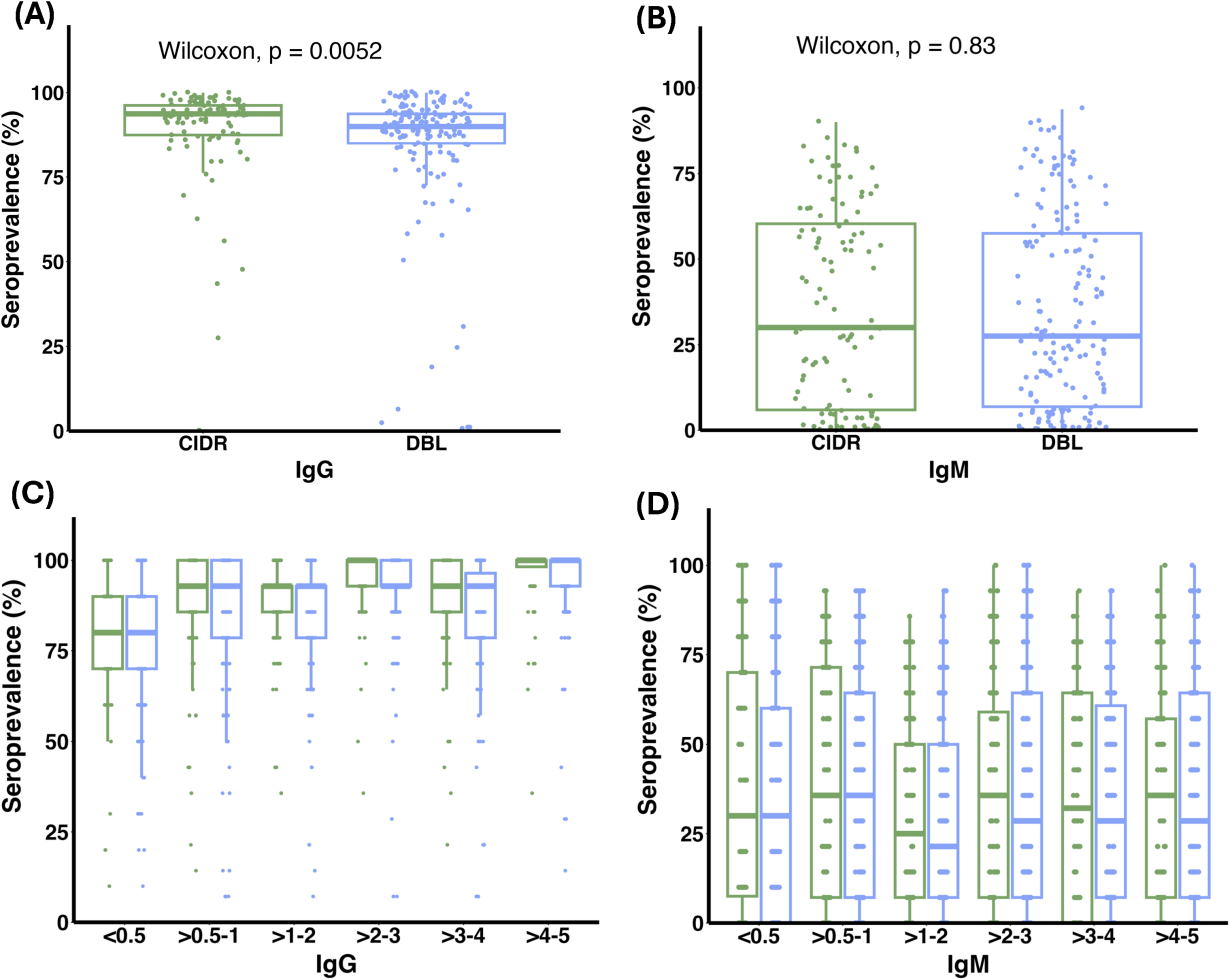
Seroprevalence of IgG and IgM antibodies to PfEMP1 domains in young children. Seroprevalence of antibodies to PfEMP1 CIDR (n=108; green) and DBL (n=163; blue) domains is shown. Box plots display medians, interquartile ranges (25^th^ and 75^th^ percentiles), and error bars representing the 10^th^ and 90^th^ percentiles. **(A)** IgG seroprevalence differed significantly between CIDR and DBL domains (Wilcoxon, *p* = 0.0052), while no difference was observed for IgM (Wilcoxon, *p* = 0.83) **(B)**. **(C, D)** Age-stratified distributions of IgG and IgM seroprevalence for CIDR and DBL domains are shown to illustrate domain-specific patterns across age groups.

To assess antigen-specific immunoreactivity, we measured IgG and IgM responses to 271 PfEMP1 domains using AlphaScreen. IgG responses were detectable against 96% of domains, and IgM against 71% (**Supplementary Table 2**), demonstrating broad early-life recognition of PfEMP1. CIDR domains showed significantly higher median IgG seroprevalence than DBL domains (median [IQR]: 93.8% [87.5-96.2%] vs 90% [85-93%]; Wilcoxon p = 0.005; **Figure 1**). When stratified by age group, IgG seroprevalence to CIDR domains was higher than to DBL domains in several age groups, with statistically significant differences observed in children aged >0.5-1, >2-3, >3-4, and >4–5 years (Wilcoxon rank sum tests, p < 0.05). By contrast, IgM seroprevalence did not differ between CIDR and DBL domains overall (median [IQR]: 30% [5.9-60.3%] vs 27.5% [6.9-57.5%]; p = 0.85). At the age-group level, no significant differences were observed between CIDR and DBL domains within any age group (Wilcoxon rank sum tests, p > 0.05 for all comparisons; **Figure 1**). These data demonstrate consistently higher IgG seroprevalence for CIDR than for DBL domains across multiple age groups, whereas IgM seroprevalence does not differ between domain classes.

### 3.3 IgG response breadth associates with age, whereas IgM breadth remains steady

**Figure 2:**
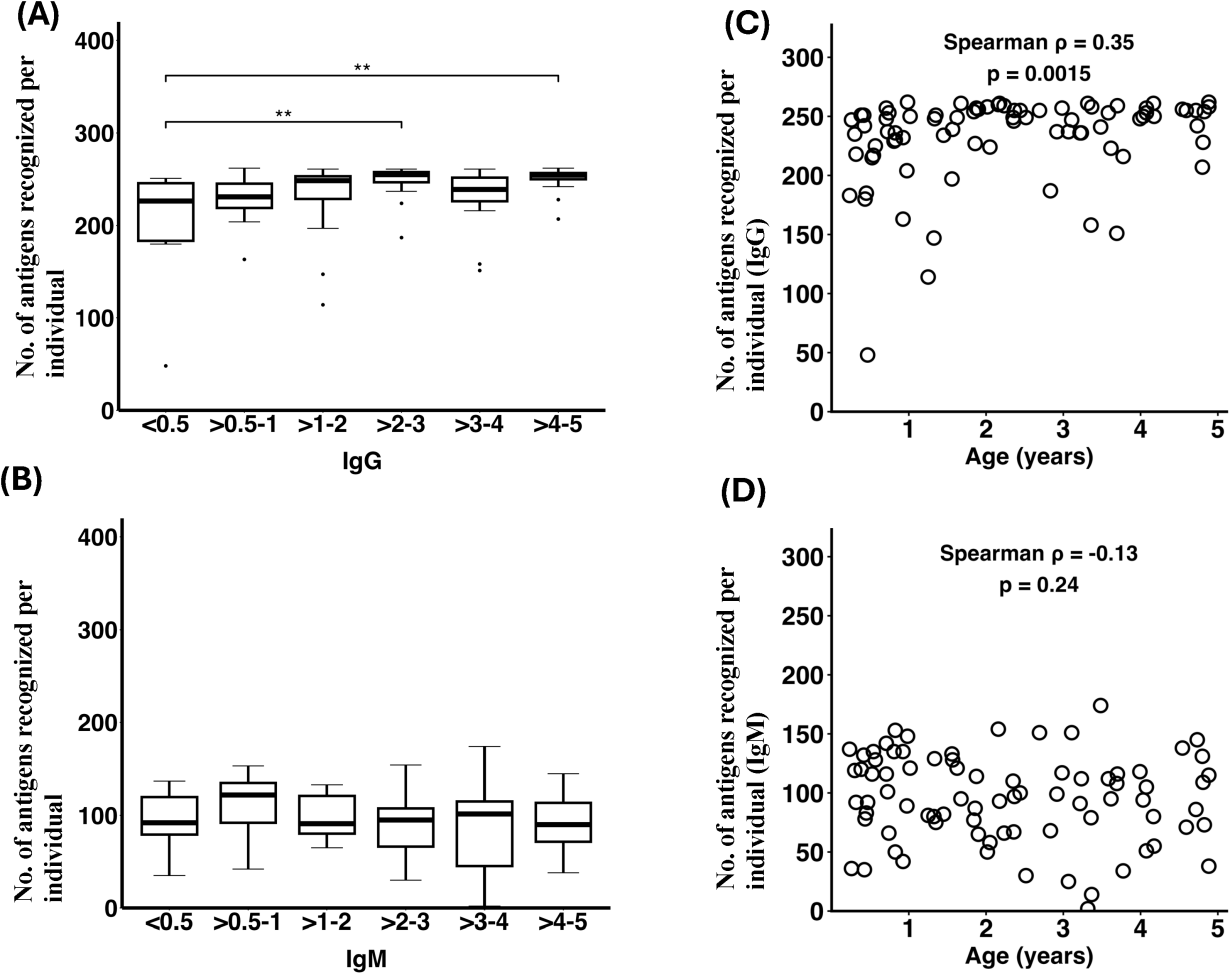
Age-dependent breadth of antibody responses to PfEMP1 domains in children under 5 years old. The breadth of the antibody response is shown as the number of PfEMP1 antigens recognized per individual across age groups. Box plots display medians and interquartile ranges. **(A)** The breadth of IgG response increased with age, with statistically significant differences observed between the youngest age group (<0.5 years) and older age groups >2–3 and >4–5 years (paired Wilcoxon rank-sum test, p <0.05, for both age groups). **(B)** In contrast, IgM antibody breadth showed no significant differences among age groups (paired Wilcoxon rank-sum test, p > 0.05). **(C)** Scatter plot showing the association between age and IgG antibody breadth, demonstrating a positive correlation (Spearman ρ = 0.35, p = 0.0015). **(D)** Scatter plot showing no significant association between age and IgM antibody breadth (Spearman ρ = -0.13, p = 0.24).

We quantified immune breadth by counting the number of PfEMP1 antigens to which each child was seropositive. The breadth of IgG response increased with age, with statistically significant differences observed between the youngest age group (<0.5 years) and older age groups >2–3 and >4–5 years (Wilcoxon rank-sum test, p <0.05 for both age groups), **Figure 2A**. The median number of PfEMP1 antigens recognized increased from 226 (IQR: 184-246) in children <0.5 years to 254 (IQR: 250-257) in children >4–5 years, **Figure 2A**. In line with this, age was positively correlated with IgG breadth (Spearman ρ = 0.35, p = 0.0015; **Figure 2C**).

IgM breadth, however, did not differ significantly among age groups (p > 0.05 for all comparisons, **Figure 2B**). Median breadth values remained comparable across age groups, ranging from 92 antigens (IQR: 79.2-120) in children aged <0.5 to 90 antigens (IQR: 71.5-114) in those aged >4–5 years. Likewise, no significant association was observed between age and IgM breadth (Spearman ρ = -0.13, p = 0.24; **Figure 2D**). These findings indicate an age-associated expansion of PfEMP1-specific IgG breadth during early childhood with no corresponding increase in IgM breadth. Whether these steady IgM responses contribute functionally to protection warrants further investigation.

### 3.4 Pre-season IgG but not IgM is widely associated with reduced malaria risk

**Figure 3:**
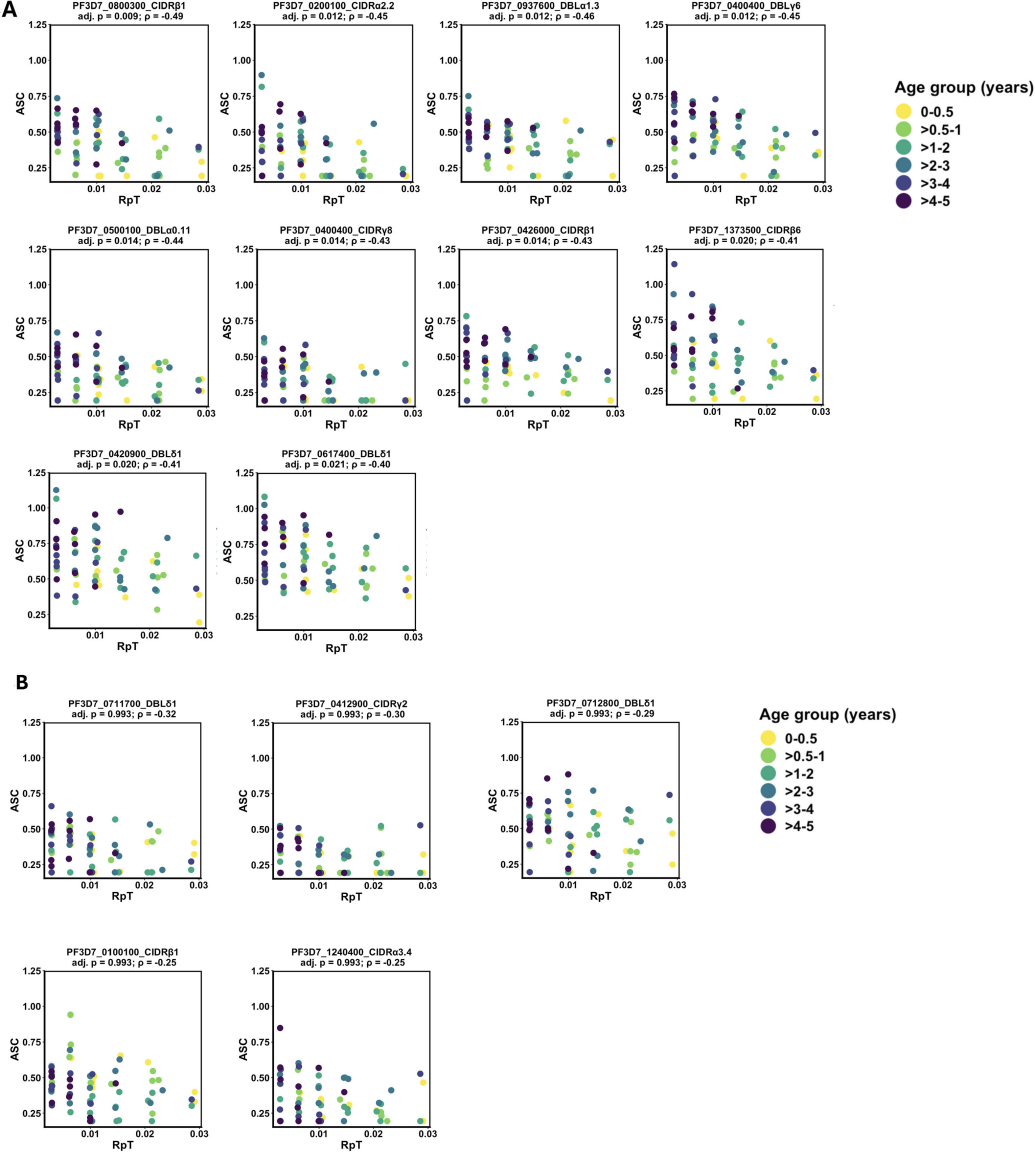
**(A)** Associations between IgG antibody responses to PfEMP1 domains and malaria risk. Scatter plots depict the relationship between normalized, log-transformed IgG antibody levels (ASC) to the top 10 PfEMP1 domains and the number of malaria episodes per risk period (RpT). Each panel is annotated with the corresponding protein ID, gene ID, and domain, together with the Spearman’s correlation coefficient and FDR-adjusted p-value. Data points are colored by age group. **(B)** Associations between IgM antibody responses to PfEMP1 domains and malaria risk. Scatter plots show the relationship between normalized, log-transformed IgM antibody levels (ASC) to the top five PfEMP1 domains and the number of malaria episodes per risk period (RpT). Each panel is annotated with the corresponding protein ID, gene ID, and domain, together with the Spearman’s correlation coefficient and FDR-adjusted p-value. None of the IgM responses remained significant after correction for multiple comparisons. Data points are colored according to age group.

To identify PfEMP1 domains for which pre-season antibody responses were associated with reduced subsequent malaria risk (ASC vs. RpT), we performed Spearman rank-correlation analyses. Among IgG responses, 170 of 271 domains showed significant negative correlations with RpT (Spearman’s r < 0 and p < 0.05). Of these, 132 remained significant after FDR correction (**Supplementary Table 3**). Thus, nearly half of the PfEMP1 panel demonstrated protective IgG associations. Based on p-value rankings, the top 10 domains (5 DBL, 5 CIDR) were selected for further analysis (**Table 2**). IgG correlation plots with data points colored by age group are shown in **Figures 3A**, with age-related ASC trends presented in **Supplementary Figure 1**. These plots demonstrate the expected age-related gradient, with older children exhibiting higher IgG responses and correspondingly lower RpT values, consistent with the progressive acquisition of immunity during early childhood. For IgM, only 5 domains (2 DBL, 3 CIDR) showed nominal negative correlations (**Table 2**, **Figure 3B**), and none remained significant after FDR adjustment.

**Table 2:** Summary of selected top significant antigens ranked by *p*-values.

Library ID	Gene ID and domain name	Seroprevalence (%)	Spearman’s Rho	Unadjusted p-value	Adjusted p-value
IgG					
DC170	PF3D7_0800300_CIDRβ1	87.50	-0.49	3.18E-05	0.009
DC12	PF3D7_0200100_CIDRα2.2	80.00	-0.45	1.39E-04	0.012
DC191	PF3D7_0937600_DBLα1.3	93.75	-0.46	1.08E-04	0.012
DC34	PF3D7_0400400_DBLγ6	92.50	-0.45	1.72E-04	0.012
DC81	PF3D7_0500100_DBLα0.11	92.50	-0.44	2.52E-04	0.014
DC37	PF3D7_0400400_CIDRγ8	73.75	-0.43	3.68E-04	0.014
DC80	PF3D7_0426000_CIDRβ1	93.75	-0.43	3.74E-04	0.014
DC273	PF3D7_1373500_CIDRβ6	95.00	-0.41	6.13E-04	0.020
DC60	PF3D7_0420900_DBLδ1	98.75	-0.46	6.59E-04	0.020
DC104	PF3D7_0617400_DBLδ1	88.75	-0.40	1.02E-03	0.021
IgM					
DC124	PF3D7_0711700_DBLδ1	70.00	-0.32	0.010	0.993
DC49	PF3D7_0412900_CIDRγ2	57.75	-0.30	0.016	0.993
DC144	PF3D7_0712800_DBLδ1	87.50	-0.29	0.019	0.993
DC4	PF3D7_0100100_CIDRβ1	10.00	-0.25	0.044	0.993
DC248	PF3D7_1240400_CIDRα3.4	21.25	-0.25	0.044	0.993

### 3.5 PfEMP1-specific antibody responses are highly coordinated across domains

**Figure 4:**
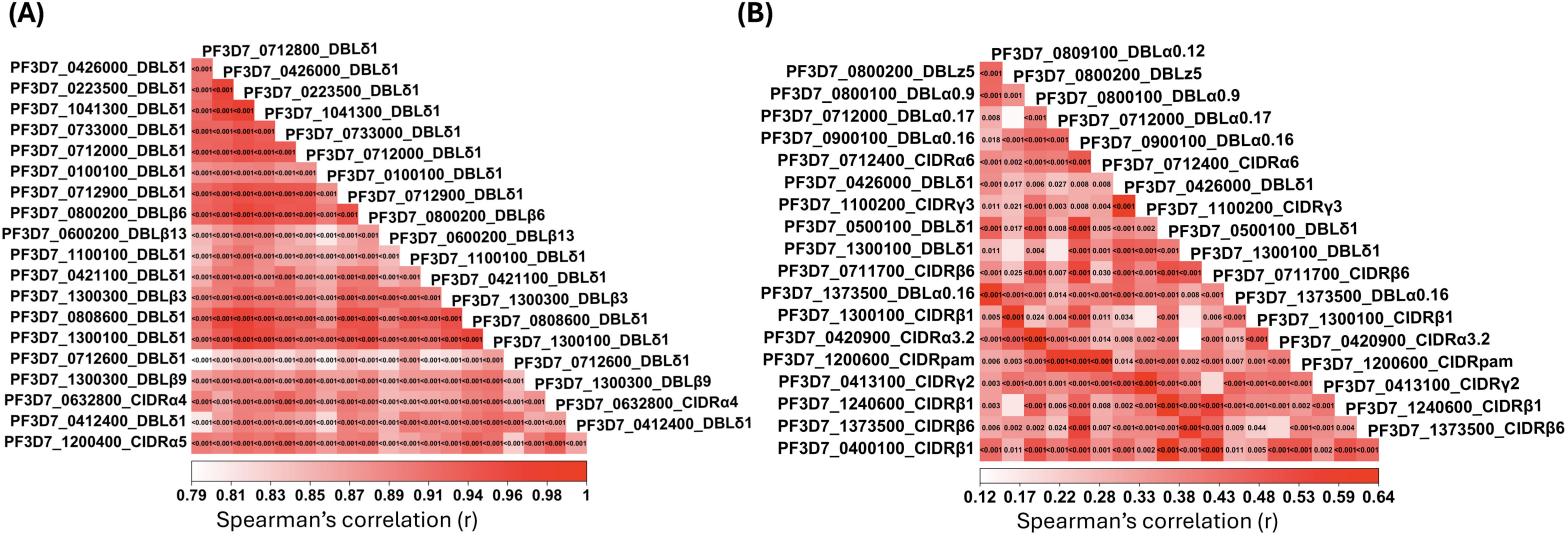
Correlation matrices of IgG and IgM responses to PfEMP1 domains at pre-season. **(A)** IgG and **(B)** IgM Spearman correlation heatmaps showing only domains involved in the strongest positive correlations. Each cell represents the correlation between two domains, with color intensity (white to red) indicating the strength of the correlation coefficient and the corresponding p-value displayed within each cell. Domains are ordered by hierarchical clustering, and full matrices including all 271 domains are shown in **Supplementary Figures 4A, 4B**.

To assess whether PfEMP1-specific antibody responses were acquired independently or in a coordinated manner, we examined interdomain correlations of IgG and IgM responses across the PfEMP1 panel. Pairwise comparisons of IgG responses showed predominantly positive correlations across domains. The median pairwise Spearman correlation among IgG responses was 0.53 (range: -0.03 to 0.97), indicating that children with high IgG responses to one domain generally also exhibited high responses to multiple domains (**Supplementary Figure 4A**). For clarity, **Figure 4A** highlights only the strongest correlations, which were predominantly observed among DBLδ1 domains. In contrast, IgM responses showed weaker inter-domain correlations overall (median Spearman’s r = 0.27; range: -0.26 to 0.64) (**Supplementary Figure 4B**), indicating a less coordinated pattern compared to IgG (**Figure 4B**). For clarity, **Figure 4B** highlights the strongest IgM correlations.

Correlations between IgG and IgM responses across domains were centered near zero (median Spearman’s r = -0.02; range: -0.48 to 0.43; **Supplementary Figure 4C**), indicating that IgG and IgM responses were largely independent.

### 3.6 Opsonic phagocytosis shows limited but domain-specific associations with protection

To assess Fc-mediated opsonic phagocytosis, we measured phagocytic activity using undifferentiated THP-1 monocytes and pre-season plasma samples. Five PfEMP1 domains representing major var groups (A, B/A, B, C) were selected from the top IgG-associated protective targets. The distribution of phagocytosis activity across age groups is shown in **Supplementary Figure 2** with summary statistics provided in **Supplementary Table 5**.

Of the five domains tested, only PF3D7_0617400_DBLδ1 showed a moderate significant negative correlation between phagocytosis and malaria risk (Spearman’s ρ = -0.26, p = 0.038; **Table 3**). The remaining four domains (PF3D7_0800300_CIDRβ1, PF3D7_0200100_CIDRα2.2, PF3D7_0937600_DBLα1.3, and PF3D7_0400400_DBLγ6) showed no significant associations (all p > 0.05). Corresponding correlation plots are presented in **Supplementary Figure 3**.

**Table 3:** Correlation between phagocytosis activity and RpT for selected PfEMP1 domains.

Library ID	Gene ID and domain name	PfEMP1 group	Spearman’s rho	*p*-value
DC170	PF3D7_0800300_CIDRβ1	B/A	0.09	0.49
DC12	PF3D7_0200100_CIDRα2.2	B	0.22	0.079
DC191	PF3D7_0937600_DBLα1.3	A/Var3	0.12	0.36
DC34	PF3D7_0400400_DBLγ6	A	0.07	0.58
DC104	PF3D7_0617400_DBLδ1	C	-0.26	0.038^*^

## 4 Discussion

In this study, we examined IgG and IgM responses to 271 PfEMP1 domains in children under five years of age living in Burkina Faso, representing, to our knowledge, the most comprehensive analysis of early-life PfEMP1-directed antibody responses to date. By measuring antibody responses at the pre-malaria transmission season using AlphaScreen, we aimed to capture early naturally acquired responses associated with reduced risk of clinical malaria. Differences in secondary detection reagents and antibody affinities precluded a direct comparison of IgG and IgM magnitudes.

We detected measurable IgG and IgM reactivity to multiple PfEMP1 domains in young children. IgG responses were broader and more widely distributed across the domain panel, whereas IgM responses were also prevalent (**Figure 1**), consistent with repeated exposure to *P. falciparum* in this high-transmission setting (38).

As maternal antibodies wane in infancy, children gradually develop their own antibody repertoires (2, 3). Consistent with this trajectory, we observed an age-related expansion in IgG breadth (0–5 years), reflecting cumulative exposure and maturation of PfEMP1-specific immunity (39, 40). However, IgM breadth did not correlate with age in our cohort. This contrasts with findings from Papua New Guinea (12), which reported age-related increases in IgM responses, but aligns with observations from Ghana (14). These differences likely reflect variation in transmission intensity, antigen panels, demographic structure, and methodology, underscoring the complexity of IgM dynamics in malaria-exposed children.

Our previous studies have consistently identified antibodies to PfEMP1 domains as correlates of clinical protection across multiple epidemiological settings (7–9). In this study, nearly half (132/271) of all PfEMP1 domains tested showed protective IgG associations after FDR correction, representing the broadest set of protective PfEMP1 targets described to date. This extensive breadth likely reflects both intense early-life exposure in this setting and the comprehensive representation of PfEMP1 domains in our panel. These data suggest that naturally acquired immunity in young children targets a broader repertoire of PfEMP1 domains than previously recognized, indicating a broad and rapidly developing antibody landscape. The strong coordination of IgG responses across PfEMP1 domains likely contributes to this pattern, as responses to many domains rise and fall together. As previously observed by Kinyua et al. (41), such coordination allows multiple antigens to appear protective in univariable analyses, functioning as correlated proxies of a shared protective immune state.

Among the top 10 protective IgG-associated domains, the highest-ranked was PF3D7_0800300_CIDRβ1, part of a DC8-type PfEMP1, consistent with findings from Kenyan children (42). Strong protective associations were also observed for adjacent DBLγ and DBLδ domains of the same protein. Although these C-terminal, non-adhesion domains are less well characterized functionally (43), antibodies to similar DBLγ and DBLδ domains have been associated with lower parasite densities in Malian children, independently of canonical adhesion regions (44). Together, these findings suggest that protective immunity to PfEMP1 extends beyond the N-terminal head structure.

Notably, most of the selected domains originated from CD36-binding PfEMP1 proteins, and several were non-adhesion domains (DBLδ, DBLγ, and CIDRβ/γ). One domain originated from a group A var3 (DC3-type) PfEMP1 with unclear receptor-binding properties (43). This distribution further supports the idea that naturally acquired protection targets multiple structural regions across PfEMP1 variants, rather than being confined to a narrow set of well-characterized adhesion domains.

In contrast, IgM responses showed weaker and less consistent associations with protection. Although five domains were associated with reduced malaria risk, none remained significant after FDR correction. Interestingly, several of these domains were also non-adhesion CD36-type PfEMP1 domains, suggesting that early IgM responses may target similar structural regions but that stable, protective associations are more clearly captured by the IgG repertoire.

Overall, these results suggest that protection against clinical malaria in early childhood is associated with a broad, highly coordinated IgG response to PfEMP1.

Finally, we assessed opsonic phagocytosis activity in a THP-1 cell-based assay for five of the most strongly protective domains. Except for PF3D7_0617400_DBLδ1, which showed a modest association with reduced malaria risk (p = 0.038), phagocytosis activity in the pre-season samples did not significantly correlate with protection. Given the broad serological associations observed, Fcγ receptor-mediated phagocytosis alone is unlikely to explain the breadth of protective antibody responses in this cohort and likely represents only one of several contributing effector mechanisms (24, 45, 46). Genetic variations in Fcγ receptor (FCGR) loci may further influence the functional consequences of these responses *in vivo* (47).

Other Fc-dependent pathways, including NK cell-mediated ADCC, may also contribute to protection (48, 49). This functional diversity likely reflects the marked antigenic heterogeneity of PfEMP1 domains, with individual domains conferring protection through distinct inhibitory or Fc-dependent mechanisms (50, 51). In addition, certain PfEMP1 domains have been shown to directly engage NK cells through interactions with NCR3 (NKp30) independent of antibodies (29, 30). Such Fc-independent PfEMP1-NCR3 interactions may help explain why protection is associated with antibody responses to diverse PfEMP1 domains, including non-adhesive regions, consistent with parallel Fcγ receptor-dependent and Fc-independent NK cell activation pathways.

Despite the limited overall contribution of opsonic phagocytosis, the domain-specific association observed for PF3D7_0617400_DBLδ1 suggests that this pathway may be relevant for selected PfEMP1 targets, consistent with previous reports (42, 43). The age range studied here (2 months to 5 years) spans a period of rapid immune maturation, including changes in antibody subclass distribution and effector cell function that shape Fcγ receptor engagement and downstream mechanisms such as phagocytosis and ADCC (52). Although antibody levels increased with age in our cohort (**Figures 3A, B**), functional maturation may be more complex and domain-specific. As a result, age-related differences in Fc-mediated effector function may have been obscured in our analyses, particularly for Fc-mediated mechanisms.

This study has a few limitations. First, the PfEMP1 panel was based solely on the 3D7 reference genome and therefore may not capture the diversity of circulating variants in Burkina Faso. Second, responses were measured at the domain level, which does not fully represent antibody recognition of full-length PfEMP1 proteins or domain architectures. Finally, due to limited plasma availability, Fcγ receptor-mediated opsonic phagocytosis was performed for only five of the ten strongest IgG-associated domains, with additional analyses ongoing. Despite these limitations, focusing on the most protective domains allowed us to identify biologically meaningful serological correlates of protection, while recognizing that other antibody effector mechanisms, such as ADCC, complement activation, or cytoadherence, may also contribute to immunity in children under age five.

### 4.1 Conclusion

This study provides the first comprehensive analysis of IgG and IgM responses to 271 PfEMP1 domains in young children living in Burkina Faso. We observed broad antibody reactivity, with nearly half of the domains showing protective IgG associations, including several non-adhesion C-terminal domains. Domain-specific opsonic phagocytosis was detected, but the findings suggest that additional effector mechanisms contribute to protection. Together, these findings highlight the breadth and heterogeneity of PfEMP1-specific antibodies as correlates of protection in early childhood malaria. Future work integrating field-variant PfEMP1, expanded functional assays, and longitudinal immune maturation tracking is warranted.

## Data Availability

The original contributions presented in the study are included in the article/**Supplementary Material**. Further inquiries can be directed to the corresponding author.
