# Supplementary material for: IgG and IgM responses to PfEMP1 domains associated with semi-immunity to clinical malaria in Burkinabe children under five
Source: Front Immunol. 2026 Apr 2;17:1781670. doi: 10.3389/fimmu.2026.1781670 (PMC13083013; doi:10.3389/fimmu.2026.1781670)
Supplement: Supplementary file 1 [file DataSheet1.zip › Data Sheet 1/SupplementaryMaterials_Frontiers/Supplementary Figures.pdf]

Supplementary Figures

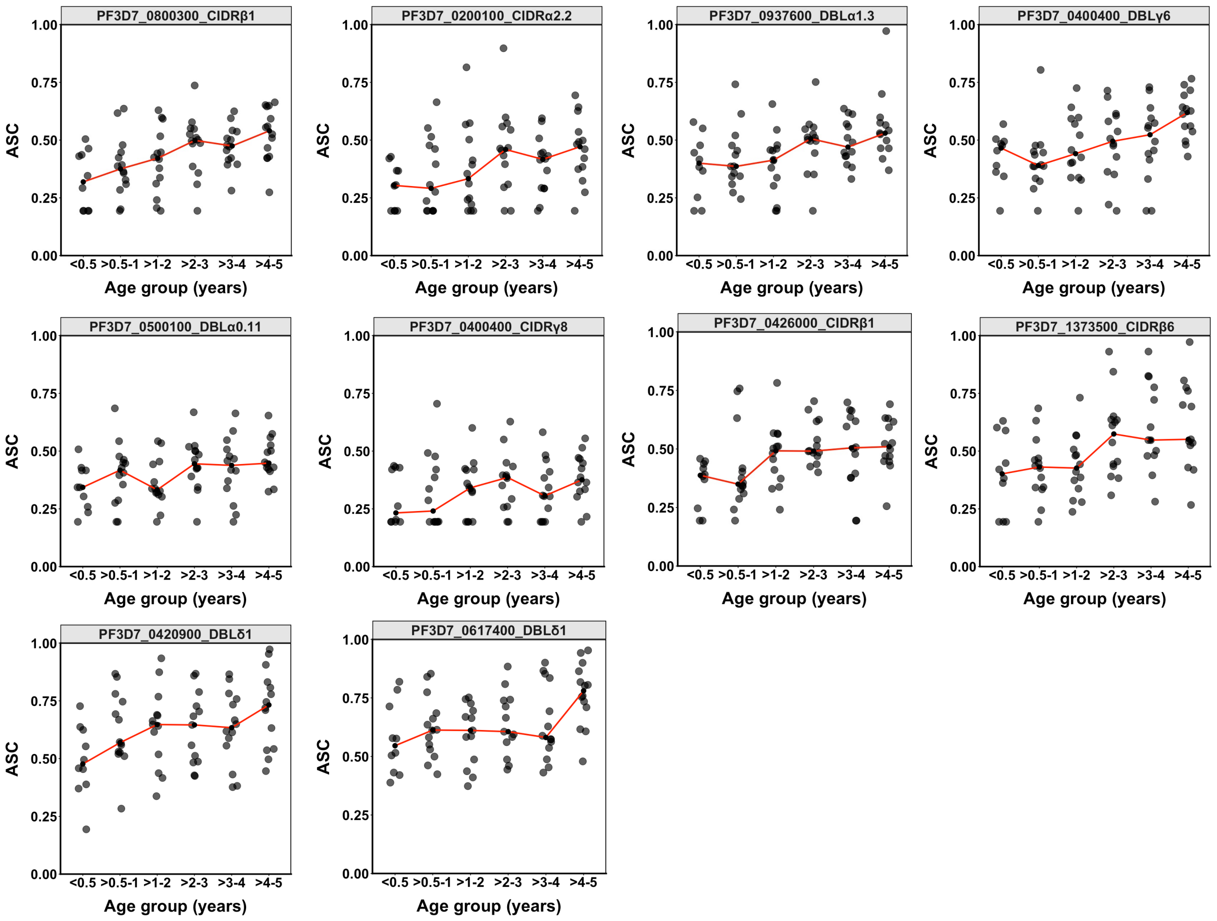

Supplementary Figure 1. Age-related changes in IgG responses to the top 10 selected PfEMP1 domains. Median IgG responses (ASC values) measured in pre-season samples are plotted against six age groups (0-0.5, >0.5-1, >1-2, >2-3, >3-4, and >4-5 years) among Burkinabe children. Each point represents an individual sample, illustrating inter-individual variability in antibody responses. Red lines represent changes in median antibody reactivity with age, highlighting age-associated patterns of naturally acquired immunity acquisition during early childhood.

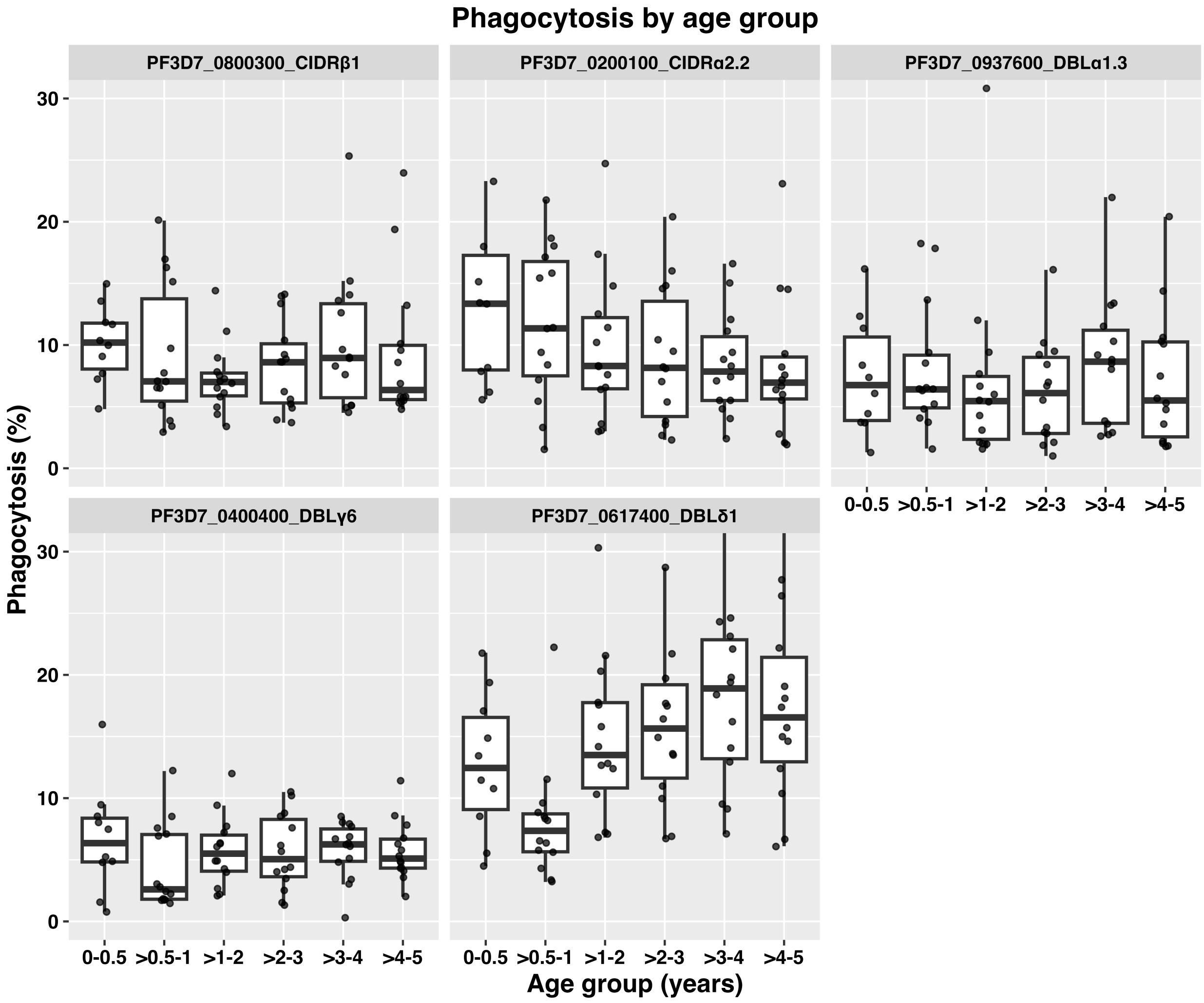

Supplementary Figure 2. Phagocytosis by age group for selected PfEMP1 domains. Box plots show the distribution of phagocytosis activity (%) across age groups for each PfEMP1 domain. Each point represents an individual plasma sample. Boxes indicate the median and interquartile range (IQR), and error bars extend to 1.5× the IQR.

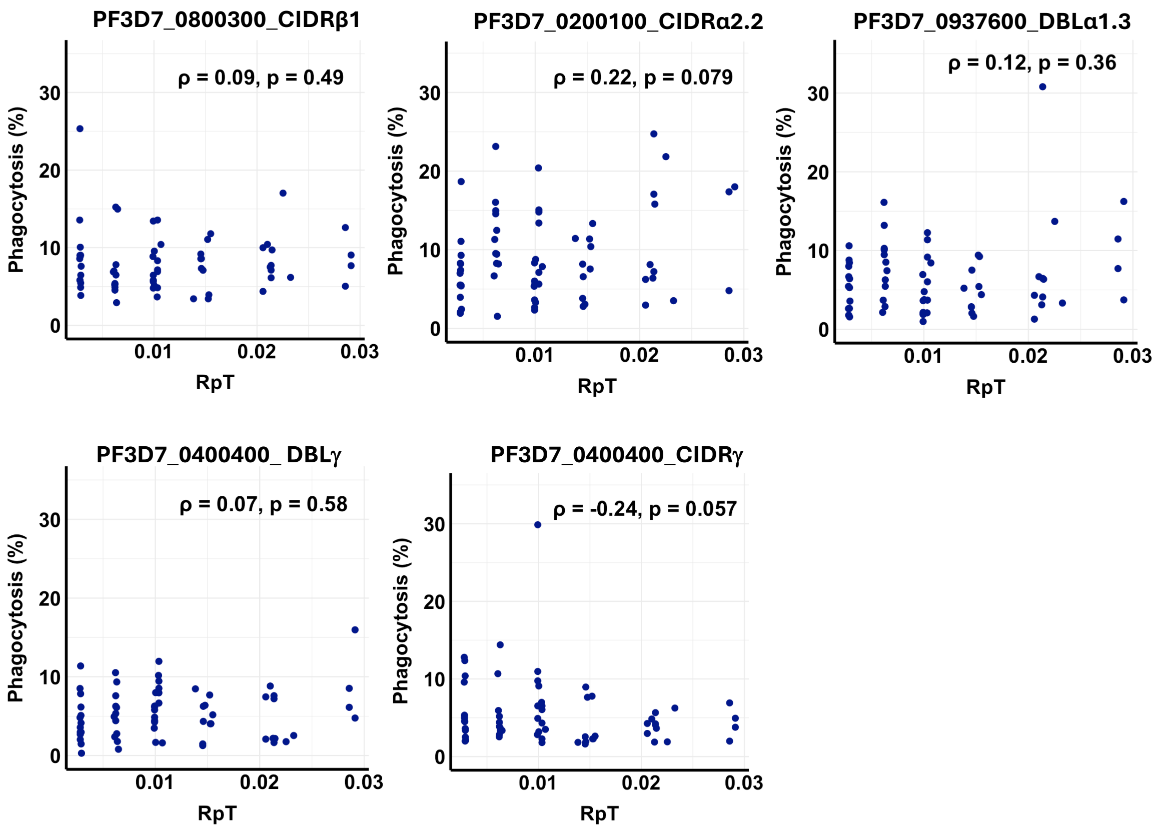

Supplementary Figure 3. Correlation between percentage phagocytosis and the number of malaria episodes per risk period (RpT) for five of the top ten selected PfEMP1 domains. Phagocytosis activity of individual plasma samples was correlated with the corresponding RpT using Spearman's correlation test. Spearman's correlation coefficient (ρ) and *p*-values are shown in each panel. Except for PF3D7_0617400_DBLδ1 (ρ= -0.26, p = 0.038), no significant correlations were observed between opsonic phagocytosis of the selected PfEMP1 domains and reduced risk of malaria episodes.

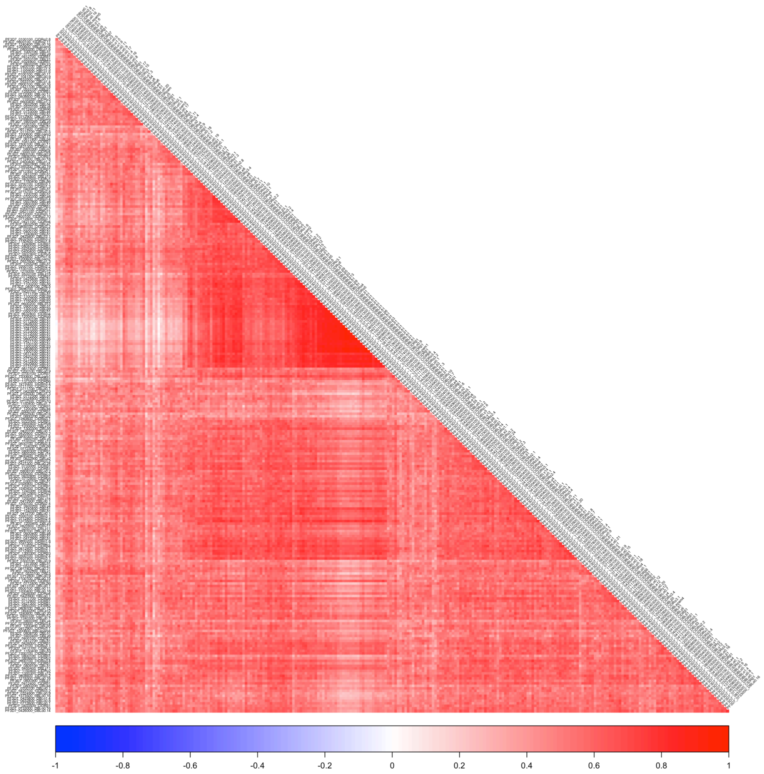

Supplementary Figure 4A. Inter-domain correlations of IgG antibody responses to PfEMP1 domains. Heatmap showing Spearman correlation coefficients between IgG responses to all 271 PfEMP1 domains across individuals. (blue, negative; white, near zero; red, positive). Domains are ordered by hierarchical clustering, and only the lower triangle of the matrix is shown for clarity. The subset of strongly correlated domains is shown in Figure 4A.

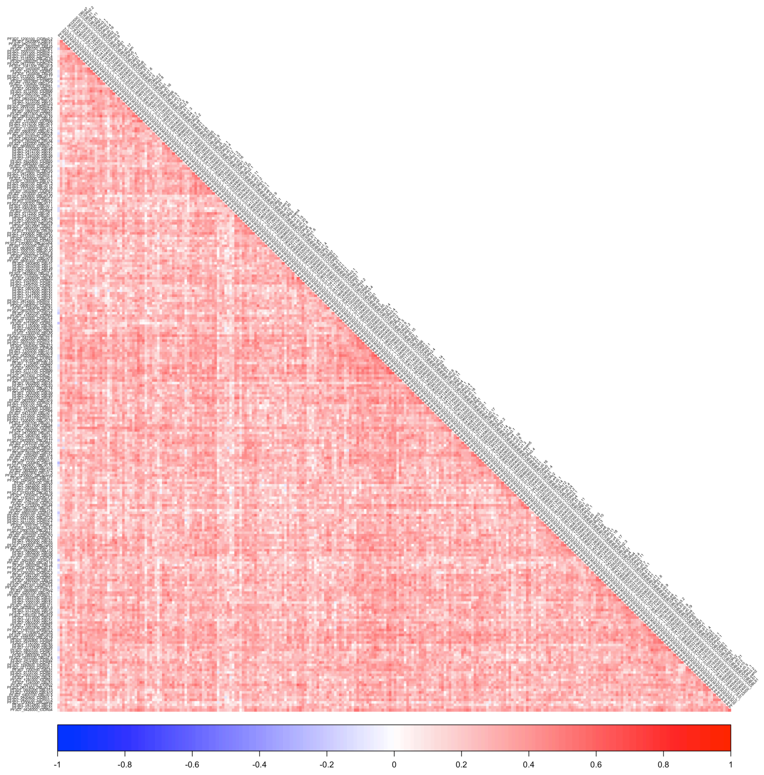

Supplementary Figure 4B. Inter-domain correlations of IgM antibody responses to PfEMP1 domains. Heatmap showing Spearman correlation coefficients between IgM responses to all 271 PfEMP1 domains across individuals. Colors indicate correlation strength (blue, negative; white, near zero; red, positive). Domains are ordered by hierarchical clustering, and only the lower triangle of the matrix is shown for clarity. The subset of strongly correlated domains is shown in Figure 4B.

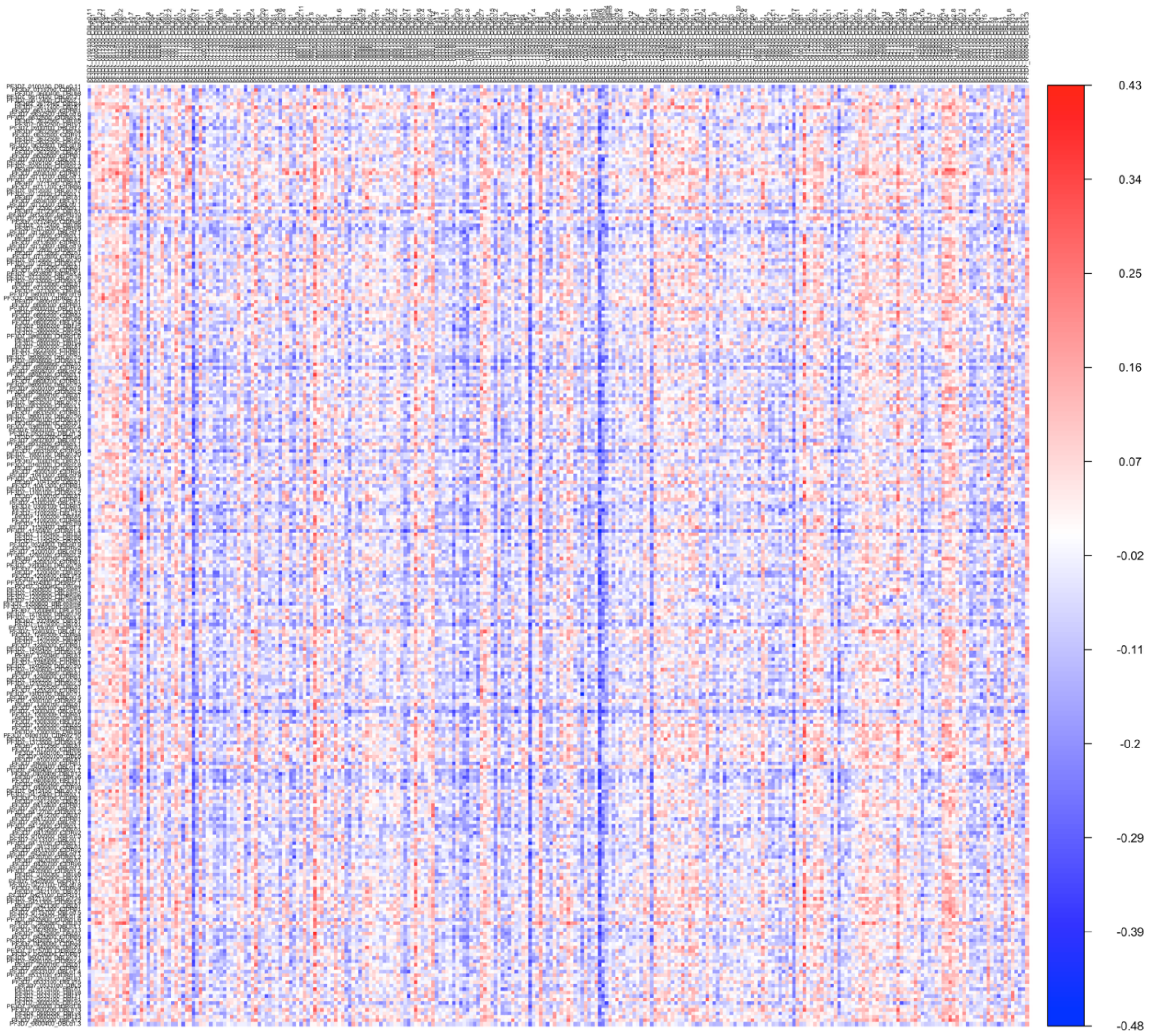

Supplementary Figure 4C. Inter-domain correlation matrix of PfEMP1-specific IgG and IgM responses. Heatmap showing correlations between IgG and IgM antibody responses to PfEMP1 domains. Each cell represents the Spearman correlation coefficient (ρ) between IgG responses to one PfEMP1 domain and IgM responses to another domain across study participants. Colors indicate the strength and direction of correlation (blue, negative; white, near zero; red, positive). Overall, correlations were centered near zero (median ρ = -0.02, IQR = 0.15), indicating limited coordination between IgG and IgM repertoires.
